# Cognitive appraisal of exposure to specific types of trauma - a study of gender differences

**DOI:** 10.1186/s12905-017-0468-x

**Published:** 2017-11-16

**Authors:** Dana–Cristina Herta, Bogdan Nemes, Doina Cozman

**Affiliations:** 1 University of Medicine and Pharmacy ‘Iuliu Hatieganu’, Cluj–Napoca, Romania; 2 Department of Medical Psychology, Str. V. Babes 43, Pavilion 3, 400012 Cluj–Napoca, Romania

**Keywords:** Nonsexual traumatic event, Posttraumatic cognitions, Gender differences, Gender specific appraisal

## Abstract

**Background:**

The role of gender in posttraumatic cognitions has increasingly been approached. The current study comparatively evaluates posttraumatic cognitions in men and women exposed to specific nonsexual trauma (motor vehicle accidents, work – related accidents, burns).

**Methods:**

Posttraumatic cognitions and posttraumatic stress symptoms were comparatively assessed in 53 men and 37 women treated in 3 Romanian primary care units after specific accidental trauma. Posttraumatic Cognitions Inventory (PTCI) was used to assess posttraumatic cognitions, and the Short Post-Traumatic Stress Disorder Rating Interview (SPRINT) was used to assess posttraumatic stress symptoms.

**Results:**

Men with significant posttraumatic stress symptoms endorsed more negative cognitions than women. Men with posttraumatic disability more consistently endorsed some negative cognitions regarding instrumentality, strength and control than their female counterparts. Women and men without posttraumatic disability reported similarly low levels of negative posttraumatic cognitions. Time elapsed since trauma increased most negative cognitions in men.

**Conclusions:**

The intensity of PTSD symptoms and presence of posttraumatic disability influence negative cognitions after exposure to accidental trauma. Women experiencing clinically significant PTSD symptoms endorse more cognitions regarding instrumentality, strength and control than male counterparts. Women with permanent disability after trauma report less cognitions involving emotionality, dependence and low self – efficacy than male counterparts. In the absence of permanent posttraumatic disability, men and women endorse similar levels of negative cognitions after accidental trauma. With time elapsed since trauma, men perceive decreasing self – efficacy, problem – solving and emotional control, while women perceive decreasing interpersonal cooperation. Despite limitations (cross-sectional design, lack of normative data for PTCI to ascertain culturally – specific gendered cognitions), this study supports the gender – sensitive approach of accidental trauma, especially when its consequences are pervasive, disabling and increasingly burdensome.

**Electronic supplementary material:**

The online version of this article (10.1186/s12905-017-0468-x) contains supplementary material, which is available to authorized users.

## Background

Posttraumatic cognitions entail sets of negative beliefs and thoughts, reflecting the individual’s distorted interpretation of a specific traumatic event. The tendency to endorse these cognitions after exposure to trauma is directly associated with the vulnerability for and intensity of posttraumatic stress disorder (PTSD) symptoms [1] and also reflects patterns of coping with trauma [1, 2]. Current studies have increasingly drawn the focus towards differences between men and women regarding trauma exposure.

While sex means being male or female biologically, gender refers to socially constructed male/female differences which lead to masculine and feminine gender roles (shared expectations of behavior given one’s gender), attitudes (views of others or situations commonly associated with one’s gender) and stereotypes (shared views of personality traits often tied to one’s gender) [3]. Some authors emphasize that differences in incidence of exposure to traumatic events in men and women may account for these differences [2, 4] and mediate posttraumatic coping [4]. Thus, men have a higher overall risk of exposure to traumatic events in general, and an increased frequency of exposure to war; women, in turn, have higher rates of exposure to sexual assault (in childhood and adulthood) and domestic violence [5]. Moreover, men and women interpret differently certain traumatic events such as childhood neglect [5], sexual abuse [6–9], nonsexual interpersonal violence [8, 10] and combat exposure [11, 12]. Also, compared with men, women exposed to trauma more often exhibit sensitivity to threats, negative misconstructions of trauma, increased willingness to verbalize/acknowledge negative emotions [8, 11, 13, 14], proneness to ruminations and depression [4, 15].

Specific types of coping strategies after trauma are ascertained [2]; some findings support that women more often endorse affiliation (interpersonal coping style) [2, 4, 16], while men more often use intrapersonal coping: self – efficacy, problem – solving and emotional control [4, 15]. Other studies show that, in military and civilian populations exposed to conflict areas, group cohesion is a stronger protective factor in men [17–19], while women exhibit increased avoidance, guardedness and startle responses, more persistent and distressing traumatic memories and less effective management of traumatic exposure [6, 20–25]. However, findings across studies do not consistently ascertain distinctive cognitive responses to trauma in men and women [26–28]. Moreover, comparisons of findings across studies are difficult due to quantitative and qualitative differences in traumatic exposure and demographic features of various studied populations [26].

Approaching posttraumatic cognitions in men and women may provide deeper understanding on the influence of gender in conceptualizing nonsexual trauma. This, in turn, could challenge gender stereotyping of posttraumatic strengths and difficulties, and generate more effective and individually tailored trauma management interventions [17, 26]. However, only one study so far comprehensively addresses gender differences in exposure to trauma and PTSD, although it mostly dwells on neurobiological factors and lacks a clear, explicit definition of gender [5]. Also, no trauma studies focused on sex or gender were performed in Romania – regardless of type of trauma exposure, assessment tool or study population.

The current study is performed in a cultural context (post – socialist North – Western Romania, large city, relatively young and educated study population) in which masculinity is conceptualized as strong, aggressive, competitive, instrumental, detached, self – reliant, invested in personal development and worker roles, while femininity is understood as effective switching of roles and behaviors between the work context (where masculine attributes are mostly expected and rewarded) and family context (where feminine is seen as passive, cooperative, expressive, submissive, nurturing, emotional, invested in others/motherhood/family) [29, 30].

The current study aims to assess gender differences in the type of cognitions reported by an adult, civilian, clinical population after exposure to accidental trauma with rates of incidence which do not directly depend on sex and a psychological impact unrelated to interpersonal violence (sexual or otherwise). Thus, the authors performed a cross – sectional comparative assessment of posttraumatic cognitions in male and female participants specifically exposed to and treated for motor vehicle accidents (MVA), work – related accidents (WRA) or burns (B).

## Methods

### Participants

The study focused on trauma with specific features – life threatening, accidental, nonsexual (without interpersonal violence expressed through sexual behaviors) and not resulting through interpersonal aggression. This inclusion criterion ensured a homogenous type of trauma exposure, without significant differences in incidence, meaning or consequences in men and women. Authors selected recruitment centers which provide comprehensive acute and follow – up medical care for adult patients exposed to trauma: the Plastic surgery and reconstructive microsurgery Clinic and the Neurology Rehabilitation Clinic of the Clinical Rehabilitation Hospital Cluj-Napoca, Romania, and the Emergency Unit of the Cluj County Emergency Hospital, Cluj-Napoca, Romania, respectively. Four undergraduate medical students from the University of Medicine and Pharmacy ‘Iuliu Hatieganu’ Cluj – Napoca, Romania, were trained and performed recruitment procedures between July 29th and December 31st 2014.

Recruiters identified 230 eligible patients exposed to life – threatening MVA, WRA or B treated in the aforementioned medical units and contacted 215 of those patients. Ninety patients (41.86% of those contacted) signed the informed consent for participation in the study; 23 participants (25.6%) received treatment in the Clinical Rehabilitation Hospital Cluj-Napoca, Romania – Neurology Rehabilitation Clinic, 25 in the Clinical Rehabilitation Hospital Cluj-Napoca, Romania – Plastic surgery and reconstructive microsurgery Clinic (27.8%), and 42 in the Emergency Unit – Cluj County Emergency Hospital, respectively (46.7%). None of the participants received psychological intervention since the exposure to trauma, all exposed to the index event at least 1 month prior to recruitment. Recruiters performed the study assessments in the recruitment sites after the patients finished scheduled follow – up assessments with the medical team in charge of their treatment, in a manner that would not interfere with the patients’ treatment.

Demographic and trauma – related data of participants were also collected (see Table 1).

**Table 1:** Demographic and clinical features of the study participants

Sample parameters	Male			Female		
Sample size	53 (58.9%)			37 (41.1%)		
Age in years^a^	36.68 (± 15.10)			31.92 (± 13.81)		
Housing	Urban 42 (79.2%)	Rural 11 (20.8%)		Urban 32 (86.5%)	Rural 5 (13.5%)	
Academic education (years graduated)	Primary (8 years) 2 (3.8%)	Secondary^b^ (12–15 years) 21 (39.6%)	Tertiary^c^ (> = 16 years) 30 (56.6%)	Primary (8 years) 2 (5.4%)	Secondary^b^ (12–15 years) 5 (13.5%)	Tertiary^c^ (> = 16 years) 30 (81.1%)
Traumatic event assessed	MVA 39 (73.6%)	WRA 13 (24.5%)	B 1 (1.9%)	MVA 31 (83.8%)	WRA 3 (8.1%)	B 3 (8.1%)
Permanent disability caused by the assessed traumatic event	Yes 31 (58.5%)	No 22 (41.5%)		Yes 16 (43.2%)	No 21 (56.8%)	
Time elapsed since trauma, in months^a^	64.75 (± 92.44)			58.92 (± 80.40)		

^a^Data presented as mean (± standard deviation)

^b^Highschool or professional schools

^c^Undergraduate and postgraduate university training

### Measures

The study used the following clinical instruments:

1. POSTTRAUMATIC COGNITIONS INVENTORY (PTCI) is a self-assessment scale of trauma – related cognitions consisting of 36 items scored on a 1 (*totally disagree*) to 7 (*totally agree*) Likert scale. Validity studies support item clustering into 3 subscales – Negative perception about self (21 items), Negative perception about others (7 items) and Self – blame (5 items), 3 items remaining non – assigned [1, 31, 32].
2. SHORT PTSD RATING INTERVIEW (SPRINT) is an 8 – item self-assessment tool evaluating the ntensity of most common symptoms following severe trauma and subsequent distress, with item scores from 0 (*not at all*) to 4 (*very much*). Psychometric studies recommend SPRINT as a versatile, brief and effective instrument for PTSD screening [33–35].

Sex was recorded based on participants’ self – assessment as male or female. Gender identity was not assessed explicitly. However, PTCI addresses attributes such as strength, dominance, instrumentality, emotional and behavioral control, detachment, self – efficacy etc. in association with traumatic exposure; therefore, using this specific assessment tool in the context of the current study can provide insight on whether such attributes are gendered in the study population after exposure to non-sexual trauma.

### Scoring and assessment procedure

After permission from PTCI and SPRINT authors was received to use the scales in the study, translation and language adaptation of scales into Romanian was performed (see Additional file 1 for Romanian version of PTCI and Additional file 2 for Romanian version of SPRINT). The reference timeframe is “the past week” for the original SPRINT; however, for the purpose of this study, participants were instructed to reference the one-month timeframe in which symptoms were most distressing.

PTCI total score was computed by dividing the sum of item scores by the number of items (36) in the scale; similarly, adding scores of items in each subscale and dividing by the number of respective items resulted in subscale scores [1, 31]. SPRINT scoring strategy provides total score as the sum of item scores [33].

Authors comparatively assessed scores for individual PTCI items, PTCI total scores and subscale scores for Negative perception about self, Negative perception about others, and Self – Blame were in male versus female participants. Also, authors compared the reported intensity of PTSD symptoms, measured through SPRINT scores, in male versus female participants.

Authors stratified the sample based on permanent disability status resulting from index trauma, with subsequent comparative assessment of posttraumatic cognitions in male versus female participants from both resulting strata (participants with and without permanent disability after trauma).

Authors identified participants as screening positive for posttraumatic stress symptoms if their SPRINT scores were 19 or higher, based on cutoffs previously validated for the Romanian version of SPRINT [35]. Authors further stratified the sample based on PTSD screening status and comparatively assessed posttraumatic cognitions in male versus female participants from both resulting strata (positive and negative screeners).

Authors further assessed the relationship between the time in months elapsed from index trauma and scores for individual PTCI items, PTCI total scores and subscale scores for Negative perception about self, Negative perception about others, and Self – Blame, respectively, in male and female participants.

Statistical analysis was performed using the IBM SPSS (Statistical Package for the Social Sciences) Statistics software, Version 22.0. T-test for independent samples and Levene’s test were employed for continuous variables in order to compare 2 independent samples and to assess equality of variances, respectively. Mann – Whitney U test was used for discrete variables, to test for differences between 2 samples. Spearmann correlation was used to test the relationship between 2 abnormally distributed continuous variables.

## Results

PTCI total scores (*p* = .170, t – test for independent samples), subscale scores for Negative perception about self (*p* = .113, t – test for independent samples), Negative perception about others (*p* = .798, t – test for independent samples), and Self – Blame (*p* = .383, t – test for independent samples), and PTCI item scores, respectively, were not significantly different in male versus female participants. Furthermore, the reported intensity of PTSD symptoms, measured through SPRINT scores, was not significantly different in male versus female participants (*p* = .467, t – test for independent samples).

Stratification based on permanent posttraumatic disability status identified in the group without permanent disability that women scored significantly higher in SPRINT than male participants (*p* = .049, Mann – Whitney U). However, male and female participants in this group reported similar scores in the PTCI total scale, subscales and items. In the group without posttraumatic disability, male participants scored significantly higher than females in 2 PTCI items: ‘I can’t rely on myself.’ (*p* = .025, Mann – Whitney U) from the Negative perception about self subscale, and ‘I will not be able to control my emotions, and something terrible will happen.’ (*p* = .008, Mann – Whitney U), non – assigned item, while similar scores were reported in all the remaining assessments.

Sample stratification into positive and negative PTSD screeners revealed that in the positive screening group males scored significantly higher than females in 2 of the PTCI items, both belonging to the Negative perception about self subscale: ‘If I think about the event, I will not be able to handle it’ (*p* = .018, Mann – Whitney U) and ‘My reactions since the event show that I am a lousy coper’ (*p* = .028, Mann – Whitney U), whileall the remaining assessments, including SPRINT, provided similar scores in male and female participants. In the negative PTSD screening group, male and female participants endorsed similar scores for PTCI total score, PTCI subscales and items, and SPRINT, respectively.

PTCI scale, subscales and most item scores were consistently higher in males from the positive screening stratum than in those from the negative one, while in female participants scores between positive and negative screening strata were consistently different in the Negative perception about self subscale scores and majority of its items, respectively (see Tables 2, 3, 4, 5 and 6).

**Table 2:** Mean scores for PTCI items from the Negative Perception about Self subscale in male versus female participants – total sample and after stratification

Negative perception about self subscale items	Positive screeners (SPRINT > = 19) *N* = 12		Negative screeners (SPRINT <19) *N* = 78		Permanent disability after trauma *N* = 47		No permanent disability after trauma *N* = 43	
	Male^a^ *N* = 7	Female^a^ *N* = 5	Male^a^ *N* = 46	Female^a^ *N* = 32	Male^a^ *N* = 31	Female^a^ *N* = 16	Male^a^ *N* = 22	Female^a^ *N* = 21
2. I can’t trust that I will do the right thing.	3.86 (± 2.85)	2.60 (± 2.60)	2.63 (± 2.00)	2.28 (± 1.67)	3.10 (± 2.25)	2.63 (± 2.02)	2.36 (± 1.94)	2.10 (± 1.57)
3. I am a weak person.	3.57 (± 2.93)	2.60 (± 1.51)	1.93 (± 1.42)	2.34 (± 1.75)	2.55 (± 2.11)	2.56 (± 2.06)	1.59 (± .79)	2.24 (± 1.41)
4. I will not be able to control my anger and will do something terrible.	3.86 (± 2.73)	1.60 (± .89)	2.07 (± 1.55)	1.63 (± 1.40)	2.81 (± 2.10)	1.75 (± 1.57)	1.59 (± 1.00)	1.52 (± 1.16)
5. I can’t deal with even the slightest upset.	4.14 (± 2.85)	2.60 (± 2.19)	2.33 (± 1.87)	1.84 (± 1.46)	3.29 (± 2.25)	2.25 (± 1.94)	1.55 (± 1.29)	1.71 (± 1.18)
6. I used to be a happy person but now I am always miserable.	4.71 (± 2.49)	4.40 (± 2.79)	2.39 (± 1.90)	1.81 (± 1.44)	3.52 (± 2.26)	3.19 (± 2.40)	1.55 (± 1.18)	1.38 (± .66)
9. I feel dead inside.	3.14 (± 2.19)	3.80 (± 2.68)	1.70 (± 1.47)	1.56 (± 1.50)	2.42 (± 1.94)	2.56 (± 2.44)	1.14 (± .46)	1.33 (± .91)
12. I am inadequate.	5.29 (± 2.62)	2.60 (± 1.67)	2.37 (± 1.85)	1.75 (± 1.36)	3.58 (± 2.39)	2.25 (± 1.77)	1.59 (± 1.09)	1.57 (± 1.02)
14. If I think about the event, I will not be able to handle it.	5.86 (± 2.19)	1.60 (± .894)	2.15 (± 1.59)	1.88 (± 1.40)	3.06 (± 2.26)	2.06 (± 1.73)	2.05 (± 1.67)	1.67 (± .96)
16. My reactions since the event mean that I am going crazy.	6.00 (± 2.23)	2.60 (± 2.60)	1.70 (± 1.50)	1.53 (± 1.29)	2.94 (± 2.42)	1.94 (± 1.91)	1.32 (± 1.28)	1.48 (± 1.16)
17. I will never be able to feel normal emotions again.	5.00 (± 2.76)	3.00 (± 2.82)	2.04 (± 1.76)	1.63 (± 1.33)	3.26 (± 2.33)	2.63 (± 2.21)	1.27 (± 1.07)	1.19 (± .40)
20. I have permanently changed for the worse.	5.00 (± 2.76)	3.00 (± 2.82)	1.93 (± 1.45)	1.88 (± 1.45)	2.97 (± 2.16)	2.25 (± 2.08)	1.45 (± 1.14)	1.86 (± 1.35)
21. I feel like an object, not like a person.	5.71 (± 2.21)	3.20 (± 2.68)	1.89 (± 1.74)	1.69 (± 1.53)	3.16 (± 2.49)	2.31 (± 2.27)	1.32 (± 1.08)	1.57 (± 1.20)
24. I feel isolated and set apart from others.	5.71 (± 2.21)	4.20 (± 2.58)	2.43 (± 1.77)	2.09 (± 1.59)	3.29 (± 2.35)	2.94 (± 2.26)	2.27 (± 1.63)	1.95 (± 1.39)
25. I have no future.	5.57 (± 2.22)	3.00 (± 2.82)	2.22 (± 1.86)	1.75 (± 1.62)	3.61 (± 2.37)	2.38 (± 2.36)	1.32 (± .89)	1.57 (± 1.24)
26. I can’t stop bad things from happening to me.	5.14 (± 2.85)	5.20 (± 2.04)	3.17 (± 2.08)	3.28 (± 2.21)	3.68 (± 2.30)	4.06 (± 2.76)	3.09 (± 2.22)	3.14 (± 1.76)
28. My life has been destroyed by the trauma.	5.86 (± 1.46)	4.20 (± 3.03)	2.26 (± 2.08)	1.72 (± 1.61)	3.74 (± 2.50)	3.13 (± 2.63)	1.32 (± 1.04)	1.24 (± .62)
29. There is something wrong with me as a person.	4.86 (± 2.73)	3.60 (± 2.07)	2.00 (± 1.66)	1.78 (± 1.40)	2.94 (± 2.33)	2.75 (± 2.11)	1.59 (± 1.22)	1.48 (± .75)
30. My reactions since the event show that I am a lousy coper.	6.00 (± 2.23)	2.60 (± 1.51)	2.17 (± 1.71)	1.91 (± 1.37)	3.32 (± 2.46)	2.19 (± 1.83)	1.77 (± 1.34)	1.86 (± .96)
33. I feel like I don’t know myself anymore.	5.71 (± 2.21)	3.80 (± 2.68)	2.04 (± 1.71)	1.66 (± 1.40)	3.39 (± 2.33)	2.63 (± 2.30)	1.32 (± 1.08)	1.43 (± .92)
35. I can’t rely on myself.	4.71 (± 2.75)	3.00 (± 2.34)	2.33 (± 1.82)	1.72 (± 1.35)	3.45 (± 2.35)	1.88 (± 1.62)	1.50 (± .85)	1.90 (± 1.51)
36. Nothing good can happen to me anymore.	4.71 (± 2.75)	2.80 (± 2.49)	1.83 (± 1.46)	1.47 (± 1.07)	2.87 (± 2.12)	2.06 (± 1.94)	1.27 (± 1.07)	1.33 (± .57)

^a^Results presented as mean scores (± standard deviation)

**Table 3:** Mean scores for PTCI items from the Negative Perception about Others subscale in male versus female participants – total sample and after stratification

Negative perception about others subscale items	Positive screeners (SPRINT > = 19) *N* = 12		Negative screeners (SPRINT <19) *N* = 78		Permanent disability after trauma *N* = 47		No permanent disability after trauma *N* = 43	
	Male^a^ *N* = 7	Female^a^ *N* = 5	Male^a^ *N* = 46	Female^a^ *N* = 32	Male^a^ *N* = 31	Female^a^ *N* = 16	Male^a^ *N* = 22	Female^a^ *N* = 21
7. People can’t be trusted.	5.29 (± 2.92)	4.80 (± 2.68)	3.80 (± 2.11)	3.31 (± 2.07)	4.35 (± 2.12)	3.69 (± 2.54)	3.50 (± 2.40)	3.38 (± 1.91)
8. I have to be on guard all the time.	5.00 (± 2.23)	4.20 (± 1.64)	4.48 (± 2.30)	4.25 (± 2.17)	5.03 (± 2.22)	4.50 (± 2.03)	3.86 (± 2.23)	4.05 (± 2.15)
10. You can never know who will harm you.	5.86 (± 2.19)	5.40 (± 1.51)	4.17 (± 2.20)	4.19 (± 2.14)	4.84 (± 2.33)	4.75 (± 2.35)	3.77 (± 2.02)	4.05 (± 1.88)
11. I have to be especially careful because you never know what can happen next.	6.43 (± 1.13)	5.00 (± 2.12)	4.63 (± 2.02)	4.69 (± 1.90)	5.48 (± 1.74)	5.06 (± 2.04)	4.00 (± 2.09)	4.48 (± 1.80)
18. The world is a dangerous place.	5.71 (± 1.79)	4.00 (± 2.00)	4.17 (± 2.06)	4.25 (± 2.00)	4.94 (± 1.89)	3.75 (± 2.23)	3.59 (± 2.13)	4.57 (± 1.72)
23. I can’t rely on other people.	6.00 (± 2.23)	4.20 (± 2.16)	2.89 (± 1.74)	3.25 (± 2.20)	3.77 (± 2.37)	4.13 (± 2.52)	2.64 (± 1.36)	2.81 (± 1.75)
27. People are not what they seem.	6.43 (± 1.51)	6.40 (± 1.34)	4.48 (± 2.03)	5.03 (± 1.84)	4.94 (± 2.12)	6.00 (± 1.75)	4.45 (± 1.99)	4.62 (± 1.68)

^a^Results presented as mean scores (± standard deviation)

**Table 4:** Mean scores for PTCI items from the Self – Blame subscale in male versus female participants – total sample and after stratification

Self – blame subscale items	Positive screeners (SPRINT > = 19) *N* = 12		Negative screeners (SPRINT <19) *N* = 78		Permanent disability after trauma *N* = 47		No permanent disability after trauma *N* = 43	
	Male^a^ *N* = 7	Female^a^ *N* = 5	Male^a^ *N* = 46	Female^a^ *N* = 32	Male^a^ *N* = 31	Female^a^ *N* = 16	Male^a^ *N* = 22	Female^a^ *N* = 21
1. The event happened because of the way I acted.	4.00 (± 2.70)	2.20 (± 1.78)	2.83 (± 2.02)	2.78 (± 2.26)	3.71 (± 2.10)	3.13 (± 2.15)	1.95 (± 1.75)	2.38 (± 2.22)
15. The event happened to me because of the sort of person I am.	4.43 (± 2.57)	2.80 (± 1.78)	2.57 (± 2.04)	2.22 (± 1.97)	3.42 (± 2.18)	3.19 (± 2.28)	1.95 (± 1.71)	1.62 (± 1.32)
19. Somebody else would have stopped the event from happening.	3.71 (± 2.69)	3.20 (± 2.68)	3.72 (± 2.12)	3.81 (± 2.32)	3.97 (± 2.12)	3.50 (± 2.36)	3.36 (± 2.25)	3.90 (± 2.36)
22. Somebody else would not have gotten into this situation.	5.00 (± 2.16)	4.00 (± 2.82)	2.91 (± 1.58)	2.69 (± 1.87)	3.74 (± 1.65)	3.44 (± 2.33)	2.41 (± 1.73)	2.43 (± 1.69)
31. There is something about me that made the event happen.	5.14 (± 2.19)	2.80 (± 2.49)	1.98 (± 1.59)	2.00 (± 1.79)	3.06 (± 2.11)	2.56 (± 2.30)	1.45 (± 1.33)	1.76 (± 1.44)

^a^Results presented as mean scores (± standard deviation)

**Table 5:** Mean scores for non – assigned PTCI items in male versus female participants – total sample and after stratification

Non – assigned items	Positive screeners (SPRINT > = 19) *N* = 12		Negative screeners (SPRINT <19) *N* = 78		Permanent disability after trauma *N* = 47		No permanent disability after trauma *N* = 43	
	Male^a^ *N* = 7	Female^a^ *N* = 5	Male^a^ *N* = 46	Female^a^ *N* = 32	Male^a^ *N* = 31	Female^a^ *N* = 16	Male^a^ *N* = 22	Female^a^ *N* = 21
13. I will not be able to control my emotions, and something terrible will happen.	4.86 (±2.73)	1.60 (±.89)	2.24 (±1.82)	1.66 (±1.20)	3.35 (±2.37)	1.50 (±.96)	1.50 (±1.05)	1.76 (±1.30)
32. I will not be able to tolerate my thoughts about the event, and I will fall apart.	4.86 (±2.73)	2.80 (±2.49)	1.87 (±1.52)	1.66 (±1.45)	2.84 (±2.26)	2.44 (±2.27)	1.45 (±1.10)	1.33 (±.57)
34. You never know when something terrible will happen.	6.14 (±1.21)	6.00 (±1.41)	4.33 (±2.24)	4.72 (±2.17)	5.13 (±2.01)	5.31 (±2.27)	3.77 (±2.28)	4.57 (±1.98)

^a^Results presented as mean scores (±standard deviation)

**Table 6:** Mean scores for PTCI scale and subscales in male versus female participants – total sample and after stratification

Scale scores	Positive screeners (SPRINT > = 19) *N* = 12		Negative screeners (SPRINT <19) *N* = 78		Permanent disability after trauma *N* = 47		No permanent disability after trauma *N* = 43	
	Male^a^ *N* = 7	Female^a^ *N* = 5	Male^a^ *N* = 46	Female^a^ *N* = 32	Male^a^ *N* = 31	Female^a^ *N* = 16	Male^a^ *N* = 22	Female^a^ *N* = 21
PTCI total	5.09 (± 1.62)	3.48 (± 1.59)	2.68 (± 1.17)	2.49 (± .98)	3.59 (± 1.53)	3.03 (± 1.44)	2.16 (± .86)	2.31 (± .64)
Negative perception about self subscale	4.97 (± 1.85)	3.14 (± 1.93)	2.17 (± 1.32)	1.86 (± 1.09)	3.18 (± 1.83)	2.49 (± 1.72)	1.62 (± .87)	1.69 (± .66)
Negative perception about others subscale	5.81 (± 1.22)	4.85 (± 1.24)	4.09 (± 1.55)	4.13 (± 1.32)	4.76 (± 1.50)	4.55 (± 1.40)	3.68 (± 1.58)	3.99 (± 1.23)
Self-blame subscale	4.45 (± 1.38)	3.00 (± 1.88)	2.8 (± 1.31)	2.7 (± 1.53)	3.58 (± 1.44)	3.16 (± 1.63)	2.22 (± .97)	2.41 (± 1.45)
SPRINT total	24.71 (± 4.07)	22.00 (± 1.22)	7.33 (± 5.05)	9.00 (± 5.05)	12.23 (± 8.07)	12.50 (± 7.16)	5.95 (± 5.44)	9.43 (± 5.80)

^a^Results presented as mean scores (± standard deviation)

In male participants, authors identified a significant direct correlation between the following parameters and time in months elapsed since trauma: PTCI total score (*p* = .017, Spearmann’s rho), Negative perception about self score (*p* = .006, Spearmann’s rho), and scores for the PTCI items 6 (*p* = .003, Spearmann’s rho), 9 (*p* = .012, Spearmann’s rho), 12 (*p* = .003, Spearmann’s rho), 14 (*p* = .010, Spearmann’s rho), 20 (p = .017, Spearmann’s rho), 21 (*p* = .003, Spearmann’s rho), 25 (*p* = .010, Spearmann’s rho), 28 (*p* = .004, Spearmann’s rho), 29 (*p* = .011, Spearmann’s rho), 30 (*p* = .018, Spearmann’s rho), 33 (*p* = .033, Spearmann’s rho), 35 (*p* = .002, Spearmann’s rho), 36 (*p* < .01, Spearmann’s rho) from the Negative perception about self subscale, and non – assigned PTCI items 13 (*p* = .038, Spearmann’s rho) and 32 (*p* = .020, Spearmann’s rho), respectively.

In female participants, PTCI item 23, from the Negative perception about others subscale, exhibited a significant direct correlation with time in months elapsed since trauma (*p* = .039, Spearmann’s rho).

## Discussions

Our study is the first to address gender differences in posttraumatic cognitions in a Romanian population exposed to accidents. Our study was implemented in a clinical setting from a metropolitan area of Romania. The relatively young study population was receiving medical care due to the enduring impact or complications of accidental trauma exposure; a subgroup of participants sustained posttraumatic physical damage severe enough to generate disability. One key finding of this study was that the intensity of PTSD symptoms and the presence of posttraumatic disability influence the level of self-reported negative cognitions after exposure to accidental trauma. Another notable finding was that this specific type of trauma exposure was associated with similar patterns of negative posttraumatic cognitions in male and female participants. Nevertheless, there were two notable exceptions to these findings. Firstly, women with PTSD symptoms warranting clinical attention from our sample reported less intense negative cognitions than male counterparts. Secondly, women with permanent disability after trauma less significantly endorsed some posttraumatic cognitions involving perceived disruption of emotional regulation, dependence on others and low self – efficacy, compared with male counterparts.

The experience of interpersonal and sexual trauma and its consequences influences women’s strategies of coping with stress and trauma [7]. The impact of sex hormones on brain development [36], social support and the influence of gender roles [37] may interact to differently shape men and women’s pathways of adaptation to interpersonal and sexual trauma [12–14, 37, 38], and may also account for sex – related differences in the development of PTSD after other types of trauma exposure [12]. Accurate event appraisal and context – adjusted behaviors and cognitions ensure effectiveness of coping to stressful events [39] and influence individual management of risk and protective factors. The cultural context plays a key role for positive and negative outcomes of coping strategies, and influences the effectiveness of coping with trauma. Coping styles which endorse affiliation, environment stability and emotion expression may be effective in youth and in social contexts fostering cohesion, connectedness and support, or detrimental in war zones, disruptive social contexts, disasters or emergencies. Conversely, social disengagement and management of emotional expression may be effective in coping with trauma exposure which cannot be influenced through active efforts to change the context, or ineffective when disengagement and efforts to control emotions could hinder the person’s access to external support [40]. Some studies ascertain gender – related factors which impact the outcome of trauma exposure [41] and include sex as predictor of physical health status after trauma [42]; however, no study so far explicitly evaluates the association between disability and cognitions related to gender role.

Other studies significantly support that sex differences related to types of traumatic exposure influence posttraumatic coping [10, 12, 43–46]; however, events assessed in those studies – sexual abuse [6, 7, 9], domestic violence [10], exposure to war [17–20] or disasters [21, 44, 47] – not only are different than those considered in our sample, but also carry a specific quantitative (different rates in men and women) and qualitative posttraumatic impact (certain types of traumatic events carry different meanings in men versus women) [2, 4, 5].

Reported intensity of PTSD symptoms was higher in women in the overall sample, negative screening stratum, and posttraumatic disability stratum, respectively, than in their male counterparts, and lower in female positive screeners than male counterparts; nevertheless, these differences were very small. In the group without permanent disability, women report significantly higher intensity of PTSD symptoms than corresponding men, in the context of similar patterns of negative posttraumatic cognitions.

The data of this study support that, in the absence of posttraumatic disability, women exposed to accidental trauma experience similar patterns of negative cognitions with men and acknowledge symptoms of psychological distress more frequently than men. These results challenge current findings that, compared with men, women are more sensitive to threats, more often misconstrue trauma negatively and, although experiencing intensities of negative emotions similarly as men, are often more willing to verbalize or acknowledge those emotions. These data, however, emerge from different populations than our research, mostly exposed to single or multiple, sexual or non-sexual, early-life or adult interpersonal trauma [10, 11, 13, 14, 48–50].

Excessive negative self – perception and self – blame identified in male participants, especially in those with levels of psychological symptoms warranting clinical attention, can also be supported through insufficient use of interpersonal coping strategies. This indirectly concurs with some findings of studies of different patterns of traumatic exposure in different populations, which identified group cohesion as a weaker protective factor for women than men, especially in contexts in which instrumentality and self – efficacy are expected from women and rewarded with group support after traumatic exposure [17–19]. Other data from civilian and military samples, however, ascertain increased avoidance, guardedness, startle responses and persistent, distressing memories and images of traumatic events in women, and also lower ability to manage traumatic events. These findings are attributed either to gender – unrelated biological factors (sensitivity to stress hormones) [6, 24, 25], or to lower beneficial effects of some protective factors that increase effectiveness of trauma management in men, i.e. women benefiting less from posttraumatic contexts in which men endorse instrumentality and self – efficacy more frequently and effectively [20–23].

Our study further shows that months unfolding since the traumatic event significantly strengthen the belief of female participants that they cannot rely on other people. Conversely, as male participants distance themselves in time from trauma, they significantly increase negative appraisals about trauma in general, and their own abilities to deal with trauma in particular, mostly expressed as lack of self – reliance, negative assessment of ability to emotionally respond and handle the impact of trauma, and negative projection in the future. These findings suggest that the gender differences proposed by our study mostly result not from traumatic exposure itself, but from the consequences and enduring impact of trauma. This perceived impact unfolding with time elapsed since trauma may reflect in multiple consequences such as impaired social functioning, protracted medical recovery, enduring physical damage or disability, ineffective coping styles. Our data suggest that the observed time dynamic of negative cognitions stems from gendered approaches in handling some perceived consequences of trauma (distress, physical damage, protracted medical recovery) and, to a smaller extent, from misconstrued interpretations of trauma (caused by low abilities of prevention and control or other negative individual features). These findings are supported by current posttraumatic resilience studies which assess perceptions of trauma as life – changing, significant, or carrying an impact on subsequent course of life events [2, 7, 43, 45–47, 50, 51].

The current study has several limitations. One important limitation is sample size. An important methodological limitation is the lack of normative data for PTCI. This limitation could be addressed by a validation study of a Romanian population exposed to accidental trauma, which could also include assessments of attitudes regarding gender role to also ascertain the validity of PTCI as assessment tool for culturally – specific gendered attitudes. Furthermore, a prospective study design with additional assessments of attitudes regarding gender role could clarify which posttraumatic cognitions are gendered and how exposure to trauma influences specific attitudes regarding gender role. Also, the study did not explicitly address coping styles and effectiveness, levels of disability and impact of disability on functioning. Assessments of the aforementioned factors in a cross – sectional or prospective design could clarify how trauma exposure, posttraumatic stress, coping and consequences of disability shape the type and level of posttraumatic cognitions.

However, this study supports that management of accidental traumatic events requires sensitivity to gender attitudes and stereotypes, especially when the consequences of exposure to trauma are more pervasive (psychological distress, impaired functioning through disability), severe and increasingly burdensome with time. A larger sample size and an additional sample of participants exposed to interpersonal trauma could provide additional data regarding the impact of trauma type on negative cognitions. A prospective research design including assessments of event centrality and coping could clarify how coping and personal meanings assigned to trauma shape negative cognitions. Further quantitative and qualitative studies of posttraumatic cognitions addressing number and types of traumatic events, posttraumatic growth and trauma centrality on a larger sample with similar demographic features and trauma exposure will provide further insight on these issues and educate both clinicians and the community on gender – sensitive approaches to accidental traumatic events.

## Conclusions

The current study supports a gender – sensitive approach of accidental trauma, especially when its consequences are pervasive, disabling and increasingly burdensome.

The study ascertains that, in the absence of permanent posttraumatic disability, men and women endorse similar levels of negative cognitions after accidental trauma. However, women experiencing clinically significant PTSD symptoms endorse more cognitions regarding instrumentality, strength and control than male counterparts. Moreover, women with permanent disability after trauma report less cognitions involving emotionality, dependence and low self – efficacy than male counterparts. With time elapsed since trauma, men perceive decreasing self – efficacy, problem – solving and emotional control, while women perceive decreasing interpersonal cooperation.

The current study also supports that the intensity of PTSD symptoms and presence of posttraumatic disability influence negative cognitions after exposure to accidental trauma.

Further studies with a prospective design, addressing culturally – specific gender attitudes, coping and perceived meanings of trauma will provide further insight on how negative cognitions are specifically shaped by the aforementioned factors and educate clinicians on individually tailored, gender sensitive trauma management.

## Additional files

Additional file 1: Romanian version of the Posttraumatic Cognitions Inventory (PTCI). (DOCX 20 kb)

Additional file 2: Romanian version of the Short PTSD Rating Interview (SPRINT). (DOCX 18 kb)

## Abbreviations

B: Burns
MVA: Motor vehicle accidents
PTCI: Posttraumatic cognitions inventory
PTSD: Posttraumatic stress disorder
SPRINT: Short PTSD rating interview
SPSS: Statistical package for the social sciences
WRA: Work – related accidents

## References

[1] Foa EB, Ehlers A, Clark DM, Tolin DF, Orsillo SM (1999). The posttraumatic cognitions inventory (PTCI): development and validation. Psychol Assess.

[2] Bosmans MW, Benight CC, van der Knaap LM, Winkel FW, van der Velden PG (2013). The associations between coping self-efficacy and posttraumatic stress symptoms 10 years postdisaster: differences between men and women. J Trauma Stress.

[3] Stets JE, Burke PJ, Borgatta EF, Montgomery RJV (2000). Femininity/masculinity. Encyclopedia of sociology.

[4] Breslau N, Davis GC, Andreski P, Peterson EL, Schultz LR (1997). Sex differences in posttraumatic stress disorder. Arch Gen Psychiatry.

[5] Olff M, Langeland W, Draijer N, Gersons BP (2007). Gender differences in posttraumatic stress disorder. Psychol Bull.

[6] Koo KH, Nguyen HV, Gilmore AK, Blayney JA, Kaysen DL (2014). Posttraumatic cognitions, somatization, and PTSD severity among Asian American and white college women with sexual trauma histories. Psychol Trauma.

[7] Shin KM, Cho SM, Lee SH, Chung YK (2014). A pilot prospective study of the relationship among cognitive factors, shame, and guilt proneness on posttraumatic stress disorder symptoms in female victims of sexual violence. J Korean Med Sci.

[8] Suliman S, Stein DJ, Seedat S (2014). Clinical and neuropsychological predictors of posttraumatic stress disorder. Medicine (Baltimore).

[9] Twaite JA, Rodriguez-Srednicki O (2004). Childhood sexual and physical abuse and adult vulnerability to PTSD: the mediating effects of attachment and dissociation. J Child Sex Abuse.

[10] Fonzo GA, Simmons AN, Thorp SR (2010). Exaggerated and disconnected insular-amygdalar blood oxygenation level-dependent response to threat-related emotional faces in women with intimate-partner violence posttraumatic stress disorder. Biol Psychiatry.

[11] Carter-Visscher R, Polusny MA, Murdoch M, Thuras P, Erbes CR, Kehle SM (2010). Predeployment gender differences in stressors and mental health among U.S. National Guard troops poised for operation Iraqi freedom deployment. J Trauma Stress.

[12] Luxton DD, Skopp NA, Maguen S (2010). Gender differences in depression and PTSD symptoms following combat exposure. Depress Anxiety.

[13] Buchanan TW, Driscoll D, Mowrer SM (2010). Medial prefrontal cortex damage affects physiological and psychological stress responses differently in men and women. Psychoneuroendocrinology.

[14] Kim SM, Han DH, Trksak GH, Lee YS (2014). Gender differences in adolescent coping behaviors and suicidal ideation: findings from a sample of 73,238 adolescents. Anxiety Stress Coping.

[15] Christiansen DM, Hansen M (2015). Accounting for sex differences in PTSD: A multi-variable mediation model. Eur J Psychotraumatol.

[16] Brown WJ, Bruce SE, Buchholz KR, Artime TM, Hu E, Sheline YI (2016). Affective dispositions and PTSD symptom clusters in female interpersonal trauma survivors. J Interpers Violence.

[17] Nash WP, Boasso AM, Steenkamp MM, Larson JL, Lubin RE, Litz BT (2015). Posttraumatic stress in deployed marines: prospective trajectories of early adaptation. J Abnorm Psychol.

[18] Woodhead C, Wessely S, Jones N, Fear NT, Hatch SL (2012). Impact of exposure to combat during deployment to Iraq and Afghanistan on mental health by gender. Psychol Med.

[19] Araya M, Chotai J, Komproe IH, de Jong JT (2007). Effect of trauma on quality of life as mediated by mental distress and moderated by coping and social support among postconflict displaced Ethiopians. Qual Life Res.

[20] Başoglu M, Livanou M, Crnobarić C (2005). Psychiatric and cognitive effects of war in former Yugoslavia: association of lack of redress for trauma and posttraumatic stress reactions. JAMA.

[21] Benight CC, Harper ML (2002). Coping self-efficacy perceptions as a mediator between acute stress response and long-term distress following natural disasters. J Trauma Stress.

[22] Gaylord KM (2006). The psychosocial effects of combat: the frequently unseen injury. Crit Care Nurs Clin North Am.

[23] Laor N, Wolmer L, Alon M, Siev J, Samuel E, Toren P (2006). Risk and protective factors mediating psychological symptoms and ideological commitment of adolescents facing continuous terrorism. J Nerv Ment Dis.

[24] Kaye JL, Dunlop BW, Iosifescu DV, Mathew SJ, Kelley ME, Harvey PD. Cognition, functional capacity, and self-reported disability in women with posttraumatic stress disorder: examining the convergence of performance-based measures and self-reports. J Psychiatr Res. 2014;57:51–7. 10.1016/j.jpsychires.2014.06.002 PMC4127348 24974001

[25] Kertzman S, Avital A, Weizman A, Segal M (2014). Intrusive trauma recollections is associated with impairment of interference inhibition and psychomotor speed in PTSD. Compr Psychiatry.

[26] Tolin DF, Foa EB (2006). Sex differences in trauma and posttraumatic stress disorder: a quantitative review of 25 years of research. Psychol Bull.

[27] Khamis V (2014). Gender responses to psychological distress among Palestinians: risk and protective factors. Br J Psychol.

[28] Lee KH, GlenMaye LF (2014). Stressors, coping resources, functioning, and role limitations among older Korean immigrants: gender differences. J Women Aging.

[29] Neculăesei A, Tătărușanu M (2008). Romania – cultural and regional differences. Analele Științifice ale Universității “Alexandru Ioan Cuza” din Iași.

[30] Verdery K, Verdery K (1996). From parent-state to family patriarchs gender and nation in contemporary Eastern Europe. What was socialism and what comes next.

[31] Beck JG, Coffey SF, Palyo SA, Gudmundsdottir B, Miller LM, Colder CR (2004). Psychometric properties of the posttraumatic cognitions inventory (PTCI): a replication with motor vehicle accident survivors. Psychol Assess.

[32] Daie-Gabai A, Aderka IM, Allon-Schindel I, Foa EB, Gilboa-Schechtman E (2011). Posttraumatic cognitions inventory (PTCI): psychometric properties and gender differences in an Israeli sample. J Anxiety Disord.

[33] Connor KM, Davidson JRT (2001). SPRINT: a brief global assessment of post-traumatic stress disorder. Int Clin Psychopharmacol.

[34] Norris FH, Hamblen JL, Brown LM, Schinka JA (2008). Validation of the Short posttraumatic stress disorder rating interview (expanded version, Sprint-E) as a measure of postdisaster distress and treatment need. Am J Disaster Med.

[35] Herța DC, Nemeș B, Cozman D (2013). Screening methodology for posttraumatic stress disorder through self-assessment scales. J Cogn Behav Psychother.

[36] Schmithorst VJ, Holland SK, Dardzinski BJ (2008). Developmental differences in white matter architecture between boys and girls. Hum Brain Mapp.

[37] Gupta A, Mayer EA, Acosta JR, Hamadani K, Torgerson C, van Horn JD, Chang L, Naliboff B, Tillisch K, Labus JS. Early adverse life events are associated with altered brain network architecture in a sex- dependent manner. Neurobiol Stress 2017;7:16-26. 10.1016/j.ynstr.2017.02.003 PMC5318542 28239631

[38] Renshaw KD, Campbell SB, Meis L, Erbes C (2014). Gender differences in the associations of PTSD symptom clusters with relationship distress in U.S. Vietnam veterans and their partners. J Trauma Stress.

[39] Folkman S, Moskowitz JT (2004). Coping: pitfalls and promise. Annu Rev Psychol.

[40] Cherewick M, Kohli A, Remy MM, Murhula CM, Kurhorhwa AK, Mirindi AB, Bufole NM, Banywesize JH, Ntakwinja GM, Kindja GM, Glass N (2015). Coping among trauma-affected youth: a qualitative study. Confl Health.

[41] Smith DL (2007). Disability, gender and intimate partner violence: relationships from the Behavioral risk factor surveillance system. Sex Disabil.

[42] Wasiak J, Lee SJ, Paul E, Mahar P, Pfitzer B, Spinks A, Cleland H, Gabbe B (2014). Predictors of health status and health-related quality of life 12 months after severe burn. Burns.

[43] Van den Broucke S, Colémont (2011). A Behavioral and nonbehavioral risk factors for occupational injuries and health problems among Belgian farmers. J Agromedicine.

[44] Zhang Z, Wang WZ, Shi ZB, Wang L, Zhang JX (2012). Mental health problems among the survivors in the hard-hit areas of the Yushu earthquake. PLoS One.

[45] Gillie BL, Thayer JF (2014). Individual differences in resting heart rate variability and cognitive control in posttraumatic stress disorder. Front Psychol.

[46] Miller ED (2015). Evaluations of hypothetical bereavement and grief: the influence of loss recency, loss type and gender. Int J Psychol.

[47] Zhou XKL, Sun X, Song H, Mao W, Huang X, Zhang Y (2013). Risk factors of mental illness among adult survivors after the Wenchuan earthquake. Soc Psychiatry Psychiatr Epidemiol.

[48] Caffo E, Forresi B, Strik LL (2005). Impact, psychological sequelae and management of trauma affecting children and adolescents. Curr Opin Psychiatry.

[49] Edmondson D (2014). An enduring somatic threat model of posttraumatic stress disorder due to acute life-threatening medical events. Soc Personal Psychol Compass.

[50] Perrin M, Vandeleur CL, Castelao E (2014). Determinants of the development of post-traumatic stress disorder, in the general population. Soc Psychiatry Psychiatr Epidemiol.

[51] Vikhireva O, Pikhart H, Pajak A (2010). Non-fatal injuries in three central and eastern European urban population samples: the HAPIEE study. Eur J Pub Health.

