# Supplementary material for: Cognitive appraisal of exposure to specific types of trauma - a study of gender differences
Source: BMC Womens Health. 2017 Nov 16;17:111. doi: 10.1186/s12905-017-0468-x (PMC5689137; doi:10.1186/s12905-017-0468-x)
Supplement: Supplementary file 1 — Romanian version of the Posttraumatic Cognitions Inventory (PTCI). (DOCX 20 kb) [file 12905_2017_468_MOESM1_ESM.docx]

INVENTARUL COGNIȚIILOR POSTTRAUMATICE (PTCI)

Ne interesează ce fel de gânduri ați avut după o experiență traumatizantă. În continuare se află o serie de afirmații care ar putea fi reprezentative sau nu pentru gândirea Dv.

Vă rugăm să citiți cu atenție fiecare afirmație și să ne spuneți cât de mult sunteți DE ACORD sau ÎN DEZACORD cu fiecare afirmație.

Oamenii reacționează la evenimente traumatizante în foarte multe feluri. Nu există răspunsuri corecte sau greșite la aceste afirmații.

*1 Dezacord total*

*2 Dezacord în mare măsură*

*3 Dezacord în mică măsură*

*4 Neutru*

*5 Acord în mică măsură*

*6 Acord în mare măsură*

*7 Acord total*

1. Evenimentul s-a întâmplat din cauza felului în care m-am comportat.
2. Nu pot să am încredere că voi face ceea ce e bine.
3. Sunt o persoană slabă.
4. Nu voi fi capabil(ă) să-mi controlez furia și voi face ceva îngrozitor.
5. Nu pot să fac față nici măcar celei mai mici supărări.
6. Eram o persoană fericită dar acum mă simt tot timpul mizerabil(ă).
7. Nu poți avea încredere în oameni.
8. Tot timpul trebuie să fiu pe fază.
9. Mă simt mort pe dinăuntru.
10. Nu poți ști niciodată cine îți va face rău.
11. Trebuie să am foarte mare grijă pentru că niciodată nu știi ce se poate întâmpla.
12. Sunt o persoană incapabilă.
13. Nu voi fi capabil(ă) sa-mi controlez emoțiile și ceva teribil se va petrece.
14. Dacă mă gândesc la eveniment, nu voi fi capabil(ă) să-i fac față.
15. Evenimentul mi s-a întamplat din cauza felului de persoană care sunt eu.
16. Felul în care reacționez de la eveniment încoace înseamnă că înnebunesc.
17. Nu voi mai fi niciodată în stare să simt emoții normale.
18. Lumea este un loc periculos.
19. Altcineva ar fi oprit desfășurarea evenimentului.
20. M-am schimbat în rău pentru totdeauna.
21. Mă simt ca un obiect, nu ca o persoană.
22. Altcineva nu ar fi ajuns în această situație.
23. Nu mă pot baza pe alți oameni.
24. Mă simt izolat și diferit de alții.
25. Nu am nici un viitor.
26. Nu pot opri lucrurile rele să mi se întâmple.
27. Oamenii nu sunt ceea ce par.
28. Viața mi-a fost distrusă de traumă.
29. Este ceva în neregulă cu mine ca persoană.
30. Reacțiile mele de la eveniment încoace arată că mă descurc jalnic.
31. Există ceva în mine care a făcut ca evenimentul să se întâmple.
32. Nu sunt capabil să-mi suport gândurile despre eveniment și o să clachez.
33. Simt că nu mă mai recunosc.
34. Niciodată nu stii când ți se va întâmpla ceva groaznic.
35. Nu pot să mă bazez pe mine.
36. Nimic bun nu mi se mai poate întâmpla.
