# Supplementary material for: Cognitive appraisal of exposure to specific types of trauma - a study of gender differences
Source: BMC Womens Health. 2017 Nov 16;17:111. doi: 10.1186/s12905-017-0468-x (PMC5689137; doi:10.1186/s12905-017-0468-x)
Supplement: Supplementary file 2 — Romanian version of the Short PTSD Rating Interview (SPRINT). (DOCX 18 kb) [file 12905_2017_468_MOESM2_ESM.docx]

**INTERVIU SCURT DE COTARE A TULBURĂRII DE STRES POSTTRAUMATIC (SPRINT)**

Vă rugăm să precizați care a fost evenimentul traumatizant care v-a afectat cel mai tare:

|  |  | Deloc | Puțin | Mode  rat | Mult | Foarte  mult |
| --- | --- | --- | --- | --- | --- | --- |
| 1 | Cât de mult ați fost deranjat(ă) de amintiri nedorite, coșmaruri sau rememorări ale evenimentului? | 0 | 1 | 2 | 3 | 4 |
| 2 | Cât efort ați depus pentru a evita să vă gândiți la evenimentul respectiv, să vorbiți despre el sau să faceți lucruri care vă amintesc de el? | 0 | 1 | 2 | 3 | 4 |
| 3 | În ce măsură ati pierdut capacitatea de a vă bucura de lucruri, ați păstrat distanța față de oameni sau vi s-a părut greu să aveți sentimente? | 0 | 1 | 2 | 3 | 4 |
| 4 | Cât de mult ați fost deranjat(ă) de probleme de somn, dificultăți de concentrare, nervozitate, iritabilitate, stare de alertă legată de ce se întâmplă în jurul Dv.? | 0 | 1 | 2 | 3 | 4 |
| 5 | Cât de mult ati fost deranjat(ă) de dureri, accese de durere sau oboseală? | 0 | 1 | 2 | 3 | 4 |
| 6 | Cât de mult v-ați supăra când vi s-ar întâmpla evenimente stresante sau ați întâlni obstacole? | 0 | 1 | 2 | 3 | 4 |
| 7 | Cât de mult au afectat simptomele de mai sus capacitatea Dv. de a lucra sau de a desfășura activitățile cotidiene? | 0 | 1 | 2 | 3 | 4 |
| 8 | Cât de mult au afectat simptomele de mai sus relațiile Dv. cu familia sau prietenii? | 0 | 1 | 2 | 3 | 4 |

Suma 1-8: _____
